# Computer-aided detection tool development for teaching chest radiograph pattern recognition to undergraduate radiography students: A context needs and capability analysis

**DOI:** 10.4102/hsag.v24i0.1322

**Published:** 2019-10-15

**Authors:** Sibusiso Mdletshe, Andre L. Nel, Louise Rainford, Heather A. Lawrence

**Affiliations:** 1 Department of Medical Imaging and Radiation Sciences, Faculty of Health Sciences, University of Johannesburg, Johannesburg, South Africa; 2 Department of Mechanical Engineering Science, Faculty of Engineering and the Built Environment, University of Johannesburg, Johannesburg, South Africa; 3 School of Medicine, Faculty of Health Sciences, University College Dublin, Dublin, Ireland

**Keywords:** computer-aided instruction, implicit skills, pattern recognition, chest radiography, simulated learning

## Abstract

**Background:**

Medical imaging (MI) education has experienced a shift aligned with the advances in technology and the role played by radiographers in pattern recognition. This has led to increased use of technology-enhanced teaching and simulated learning approaches (e.g. computer-aided detection [CAD] tools) which also support the increasing requirement to develop pattern-recognition skills at undergraduate level. However, the development of these approaches need to be explored and planned carefully to be context-relevant.

**Aim:**

The aim of this study was to explore and describe the need for and capability of a CAD tool for teaching chest radiography pattern recognition in an undergraduate radiography programme.

**Setting:**

The setting was a university that offers MI education.

**Method:**

The study employed a qualitative descriptive design with an interpretive research paradigm. Purposive sampling was used to recruit information-rich participants for a focus group interview. Information-rich participants were considered to be those who were involved in teaching clinical skills, such as those required in pattern recognition, to radiography students. Data were transcribed verbatim and analysed in a step-by-step approach.

**Results:**

Three main themes emerged: (1) a structured approach to enhance implicit skills is critical in the CAD tool design; (2) an authentic tool which is able to simulate real-world experiences in image analysis is essential; and (3) a tool which encourages self-directed learning using a wide variety of pathological conditions would be ideal.

**Conclusion:**

The results of this study are essential in guiding radiography educators in designing CAD tools for teaching chest radiography pattern recognition.

## Introduction

The increasing role played by radiographers in pattern recognition, coupled with the advances in technology, requires a shift in the teaching of radiographers at undergraduate level (Lindner 2017; Malathi et al. 2016; Shanahan 2016). As early as in 2003, higher education institutions were already reporting the introduction of basic image interpretation skills into the skill set required at undergraduate level (The College of Radiographers 2003). In 2012, The College of Radiographers issued a position statement which concluded that preliminary clinical evaluation by radiographers must be a part of the ongoing patient management and it should be taught at undergraduate level (Society of Radiographers 2013). This evaluation involved pattern recognition of radiographs. The situation is not dissimilar in the South African context, where during undergraduate studies, students are taught basic pattern-recognition skills.

In this study, we concentrated on pattern recognition of chest radiography because it is one of the commonly requested examinations in radiography because it serves as a good initial examination for a number of diseases (Eisenberg & Johnson 2012; Lisle 2012). Chest radiographs are therefore the most common radiographs that students review during their training. Piper et al. (2014) also reported that in the UK imaging departments, general radiographic examinations accounted for 62% of imaging studies and approximately 20% were of the chest. The role played by plain chest imaging has led to an increase in the emerging role played by radiographers in reporting these radiographs (Brealey et al. 2005) and therefore requires teaching approaches that enhance the development of the pattern-recognition skills for this anatomical region.

Computer-aided detection (CAD) tools are amongst the tools that could be used for the approaches that are aimed at enhancing the development of radiography pattern-recognition skills within a simulated environment (Paiva & Prevedello 2017). The development of CAD tools must address context practice and education needs.

The development of context-relevant CAD tools to teach students pattern-recognition skills is very limited. It requires an exploration of what such tools should be capable of teaching to ensure their efficient design. However, there are no South African studies to date that have been conducted to explore the needs for and capabilities of CAD tools so that they may be designed effectively. There are also no publications on the needs for and capabilities of CAD tools designed for teaching chest radiography pattern recognition. Hence, the study aimed to explore and describe the needs for and capabilities of CAD tools for teaching chest radiography pattern recognition in an undergraduate radiography programme.

## Definition of concepts

### Computer-aided detection tool

The class of computer systems that assist in the detection and/or diagnosis of diseases through a ‘second opinion’ with a goal to improve the accuracy of image reviewer and reduction of time in the interpretation of images (Firmino et al. 2016).

### Pattern-recognition skills

Pattern-recognition skills may be defined as the ability to recognise normal anatomical and physiological appearances on an image and the variations in appearances which may indicate pathology (Corr 2001).

### Implicit knowledge

It is the knowledge that can be learnt by the process of repetitive stimulus-response binding approach and leads to the development of implicit skills, that is skills that are learnt incidentally and through repetitive stimulus-response binding (Chen et al. 2017).

## Method

### Study setting

The study was undertaken in the Department of Medical Imaging and Radiation Sciences (MIRS), University of Johannesburg. This institution has an annual first-year radiography intake of 95 students for a 4-year bachelor’s degree.

### Research design

A qualitative, exploratory, descriptive design was relevant for this study as the study focused on exploring and describing the need for and capabilities of CAD tools from an end-user perspective. The qualitative descriptive design allowed the participants to describe in words what they considered to be the needs for and capabilities of CAD tools based on their experiences (Bricki & Green 2007). The participants were encouraged to convey their ideas based on their experience of being practising radiographers and their involvement in teaching clinical skills, inclusive of chest radiography pattern recognition, to undergraduate radiography students.

The focus group with purposefully selected participants was chosen as a method of data collection because the researcher sought to understand the group’s views and experiences in chest pattern recognition rather than an individual’s experiences. In addition, interviewing is particularly useful as a research method for assessing experiences and perceptions which could be difficult to capture in a formal questionnaire. This approach also allows the researcher to directly obtain insider’s view and observe the situation and behaviour (Holloway & Galvin 2017).

A single focus group interview was deemed sufficient because the participants were considered to be rich in information. Guest, Namey and McKenna (2016) have shown that at least 60% (two-thirds) of the data are obtainable from the first focus group interview. In addition, in the context of this study, the focus group interview was used as an exploratory measure to achieve incremental improvements in artefact design (Stewart, Shamdasani & Rook 2007), because the data obtained from the focus group were going to supplement the available literature on the issues related to software design for pattern recognition in Medical Imaging (MI). The study also adopted the notion of balancing resources and the fact that expert participants are difficult to be found (Stewart et al. 2007).

The focus group interview was based on guidelines from Gilliland (2014) regarding the interview process, which is an art that can assist in yielding results with objectivity. These guidelines consider the following:

- Researcher situation.
- Minimisation of social dissonance: this was assured by interviewing participants of varying age, race and educational status and from both private and public work-based learning placement (WBLP) sites.
- Use of appropriate interviewing techniques to elicit detailed views or opinions.
- Maintaining confidentiality whilst treating all participants with respect within a safe and secure environment.

### Study population and sampling

The study participants were practising radiographers who fulfil the role of student training on WBLP sites (clinical tutors). The clinical tutors were considered to be rich in information because, amongst their responsibilities, they are capable of teaching pattern recognition during WBLP. Different radiology departments form principal WBLP sites for radiography students. Students are supervised by clinical tutors and other qualified radiographers employed at each WBLP site. The MIRS Department had an advisory group comprising clinical tutors from different WBLP sites. The advisory group meets once per term (every 3 months) and each meeting is attended by a minimum of 25 clinical tutors. Recruitment of participants was carried out during one of the advisory group meetings where the researcher explained the aim and objectives of the study. At the conclusion of the meeting, volunteers were given an opportunity to be enlisted to participate in the study.

### Data collection

Data were collected by the researcher through a focus group interview. The interview took place on the day of advisory group meeting, avoiding the participants travelling on any other day. The participants were asked two fundamental questions in the interview:

1. What do you consider to be the needs for CAD tools in pattern recognition of the chest?
2. How could such a tool be useful in the training of radiographers?

The researcher then used interviewing techniques (including confirmation, probing and paraphrasing) to gain detailed views or opinions. The focus group interview lasted for about 50 min and the researcher recorded field notes which were used to support the emerging themes and categories. After transcription of the interview, the participants were given the opportunity to confirm its accuracy (data verification).

### Data analysis

**FIGURE 1:**
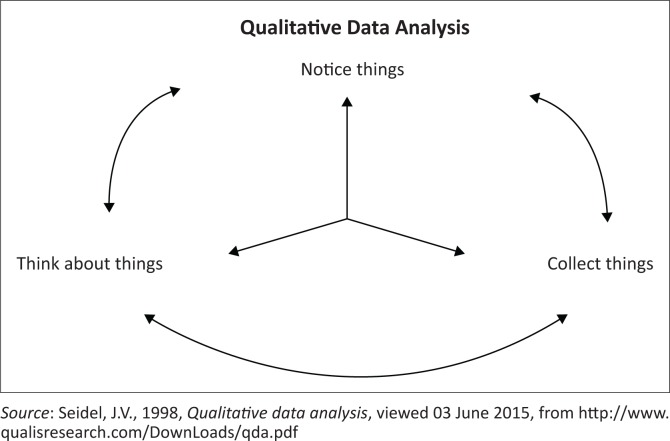
Data analysis process.

The focus group interview was audio-taped and later transcribed verbatim by a professional transcriber. This was followed by data analysis using qualitative data analysis approach. The transcribed data were read to obtain a general sense of information, which was followed by data coding. Themes were generated from the codes and the data were interpreted to generate user input (Creswell & Clark 2007). Quality data analysis was performed as described by Seidel (1998) based on noticing, collecting and thinking about things. It was a nonlinear model, and the process could move back and forth between these three aspects, as shown in Figure 1.

According to Seidel (1998), the model has the following characteristics:

- iterative and progressive, that is, it is a cycle that keeps repeating
- recursive, that is, one part can call you back to the previous part
- holographic, that is, each step in the process contains the entire process, for example, whilst noticing you are already mentally collecting and thinking about things.

Noticing involved taking field notes and ensuring that the focus group interview was recorded. Noticing progressed to colour coding of themes within the transcribed data. Whilst coding, observations were made as to the category of the question to which the codes belonged. This was completed to have a general feel that both questions of the interview were answered. Collecting and sorting out things followed, which Seidel (1998) describes as similar to fitting the pieces of a puzzle together. The identified themes were grouped into categories as part of the collecting and sorting process. The analysis concluded with thinking about things, which entailed a reflection on the nature of discoveries and how these fit in with the project.

### Ethical consideration

Ethical approval to undertake the research study was granted by the Academic Ethics Committee of the University of Johannesburg (AEC64-01-2013). The four principles for resolving ethical considerations (respect for autonomy, non-maleficence, beneficence and justice) were applied throughout the study (Dhai & Mason 2011:43–44).

## Research findings and discussion

A total of eight radiographers participated in the focus group interview. The gender split of the focus group was 50% females and 50% males, that is four females and four males. The average age of the participants was 42 years, with four participants in the age range of 30–39 years and the remaining in the age range of 50–59 years.

Five participants (62%) held a Bachelor of Technology (BTech) in Radiography as the highest qualification and three (38%) were in possession of a National Diploma in Radiography. Six participants (75%) were not enrolled for any formal qualification during the period of interviews and two (25%) were enrolled for qualifications not aligned with radiography. Over 85% of the participants had more than 5 years post-qualification experience, and 50% had more than 20 years of experience (Table 1).

**TABLE 1:** Clinical experience of the focus group participants (*n* = 8).

Variables	Year	*n*	%
**Clinical radiography experience**	0–4	1	12.5
	5–9	1	12.5
	10–14	2	25.0
	15–19	0	-
	> 20	4	50.0
**Total**		**8**	**100**

The following three themes emerged from data analysis:

1. A structured approach to enhance implicit skills is critical in the CAD tool design.
2. An authentic tool which is able to simulate real-world experiences in image analysis is essential.
3. A tool which encourages self-directed learning using a wide variety of pathological conditions would be an ideal tool.

For easy reading, participants were coded as PTCP, and M/F represent the gender of the participant.

### Theme 1: A structured approach to enhance implicit skills is critical in the computer-aided detection tool design

The participants highlighted that the pattern-recognition skills in students were lacking and in some cases students did not understand the difference between pattern recognition and image evaluation. They indicated that there was a need to develop alternative approaches to enhance teaching pattern recognition in students:

‘Because the student’s knowledge of pattern recognition is shocking. They have just come to this stage where I just do the image, it kind of looks like a chest, let’s go …’ (PTCP 2M)

‘What I’ve also come to notice is … sorry, sorry … is the student don’t know the difference between pattern recognition and film evaluation. They don’t know the difference between those two. So if a tool like that can come into practice and into play then it will be easy *to* distinguish between the two because if I evaluate a student, *she* goes the trachea and say it is symmetrical, blah-blah-blah, there’s no rotation. I’m doing pattern recognition, you are evaluating the film now. Come, tell me about densities, tell me about … they don’t know the difference.’ (PTCP 4F)

The need for a paradigm shift in both medical and MI education to align with the current developments in technology has been highlighted by several authors (Lindner 2017; Malathi et al. 2016; Shanahan 2016). The paradigm shift requires the use of alternative approaches to enhance students’ implicit skills, e.g. pattern-recognition skills. Amongst the alternative approaches to support the paradigm shift is the use of virtual teaching tools, including the use of technology-based teaching and immediate automated feedback as a reinforcement process (Chen et al. 2017). The development of CAD tools would be one of the ways to adopt approaches that enhance the implicit skills of students.

The participants were of the view that CAD tools could assist students in developing problem-solving skills and in making associations with what they see displayed. The participants pointed out that the problem-solving skills would assist students in making association with the patterns they see with the presentation of patient and thus enable them to contribute to the management of patient, for example by performing further views to better evaluate the suspected condition or pathology:

‘And the other thing that I picked up last time when we *were* doing the critical review on the patterns was there *was* certain description that you can give. You can really know as a third-year student that this can be related to A, B, C, D. They know how to do pattern recognition for certain types of pathology they know, but some of the … or that they do pattern recognition they can tell that this can be related to this, not actually diagnosing, saying knowing that pneumonia may be started at the lower [INDISTINCT] so this can be related to … it will also [INDISTINCT] with that critical thinking to say once they’ve seen different patterns they’ll know at some point that it could be associated with [INDISTINCT].’ (PTCP 6M)

‘They don’t look for problems anymore, they don’t look at their image to say they questioned this but maybe that could be another underlying problem.’ (PTCP 2M)

The CAD tools, which are a form of simulated learning, allow students to practice certain skills in safe environment (Shanahan 2016), and as such could be an ideal platform to teach students problem-solving related to pattern recognition. The use of CAD tools could enhance their pattern-recognition skills by enhancing their ability to associate certain patterns with particular diseases.

The participants further highlighted that a CAD tool could teach students a structured approach to pattern recognition. The structured approach would assist students in knowing where to start in reviewing the image and how to go about doing it systematically. Such a structured approach is critical for developing implicit skills as it is an effective method for reinforcement (Chen et al. 2017). The participants clearly indicated that the approach should allow reinforcement:

‘Anyway, she used to tell us that if you start doing a pattern recognition, you should have a pattern. Either start at the spine *or* the chest, for argument sake, go outwards or start from out and go inwards. So I think …’ (PTCP 4F)

‘So you are saying it must have a structured approach?’ (PTCP 1M)

‘Yes.’ (Group).

‘It mustn’t allow anybody just to start from the top.’ (PTCP 4F)

The structured approach harmonises well with the use of simulated learning (including CAD tools) as highlighted by Lateef (2010) and Shanahan (2016). Simulation allows students the following:

- to repeat activities until satisfied with the results (repetitive practice)
- to quickly see images and understand whether changes are to be made (feedback)
- purposeful implementation, which helps students develop technical and cognitive skills.

The development of CAD tools as a form of simulated learning was therefore highlighted as a critical component for the use of structured approaches that could enhance implicit skills in students.

### Theme 2: An authentic tool which is able to simulate real-world experiences in image analysis is essential

The participants expressed the need for a CAD tool to enhance the observational skills of students whilst sharpening their ability to pick up the abnormal findings easily:

‘And it will increase their observing skills ‘cause immediately you see it, you just look for this and that and that and that.’ (PTCP 3M)

‘Your eye will be trained, basically to identify the abnormal patterns.’ (PTCP 3M)

The participants further stated that the CAD tools could be an ideal platform for students to engage with similar images repeatedly until they are perfect in that particular skill, a view also expressed by Lateef (2010) and Shanahan (2016). This could be linked to the fact that all images would be available on one platform without retrieving them from different platforms or placing them in a viewing box:

‘So the last one at the end after seeing most of them he could answer most of the questions. So having to see a thing again, it will also help you to have an overall knowledge before you can even go and do that pattern recognition. I think let’s say if I had to take a third year … ‘cause this was a first, if I had to take a third year, I think by the time they are … they have viewed the system maybe several times, but I think they’ll be at that level.’ (PTCP 6M)

‘Interaction with the tool will help to increase their knowledge, so the need for the tool is to assist with increasing the knowledge base because the more you interact, the more it’s … okay.’ (PTCP 1M)

The other aspect regarding the authenticity of CAD tool is its performance. For example, the participants considered that it was important for the CAD tool to have good resolving ability, that is good resolution. They deemed important a high standard of image resolution, as a practitioner could miss important findings of image without good resolution:

‘I don’t know if I’m … I’m starting the second one. From my experience, what I’ve seen, I’m thinking the resolution, since it might be computer-related. The resolution is very important since every time when we view an image you might get the incorrect information ‘cause of the resolution of the image. Having what we CR I’ve seen that you can do only work with thicker areas like pelvis, like when you do oblique, you find that in other brands … I won’t mention names, in other brands you get a good resolution on a thick … on a thicker part like pelvis and then with other equipment you get grey images. So that can lead to … in terms of pattern recognising to identifying something that is not there, taking it for something else. So I think if you gonna use a tool that’s gonna have images, it must have a good resolution. So if they have to invent something they must look at the quality also.’ (PTCP 3M)

Resolution is an important part of image display in MI as it can affect the level of accuracy for the review of image. In the context of digital radiography, the spatial resolution is inversely proportional to the pixel size and is also affected by the matrix and image receptor size (Carlton & Adler 2006). The CAD tool should thus be able to consider display conditions in order to ensure that the resolution of the image is maintained. The CAD tool thus designed should not result in any loss of image resolution.

Additionally, the participants insisted that the CAD tool must perform consistently, which helps to maintain quality of the image:

‘I think I concur with the two colleagues on the part where we talk about image resolution of quality ‘cause I was coming to say, we need to have a system which will provide us with consistency in terms of showing us … of giving us the adequate quality of the image, and I’m speaking in terms of your exposures, what you expose or whatever exposure factor you said, we should get exactly the same image on the screen. You need not need to be adjusting or do as much post-processing as what we see happening currently because if you are doing pattern recognition and you fiddle too much where there … that’s where you may lose certain things and get into misdiagnosis.’ (PTCP 2M)

‘… So it should perform consistently.’ (PTCP 1M)

Medical imaging equipment is manufactured by different manufacturers. The participants reported that the CAD tool must be able to work with images irrespective of their source, that is with various equipment brands. They also highlighted that the artefact should have the ability to function with images generated from different equipment brands without losing any quality or resolution of the image. This is a critical aspect because the developed tool should have the ability to upload images irrespective of their source. This view was based on participants’ experience of having seen the image appearance changing if uploaded on a different platform:

‘I think if we go back to that … after I asked you that question, in my experience, I’ve come across situations where on one computer it’s one make and then you got a different make. So the image that you get from that make and if you transfer it to another computer that’s a different make, the quality … the resolution … the densities change. So that’s one little aspect that we need to look at just to … I think that’s gonna be a little bit of a pitfall if you gonna have two computers.’ (PTCP 4F)

‘But now I think it must highlight the issue of working with different computer systems.’ (PTCP 5F)

‘So we should be saying that it should offer consistency within adaptable …’ (PTCP 3M)

Medical imaging equipment is designed to operate within the Digital Imaging and Communications in Medicine (DICOM) standard, which is a computer software standard that allows different digital imaging software to understand each other (Carlton & Adler 2006). The designed CAD tool therefore needs to work with the DICOM standard-generated data.

The participants were of the view that the CAD tool must have the ability to perform measurements as part of its authenticity. The ability to perform measurements in pattern recognition is important because it may help radiographer to determine the density and size of structures, and thus be able to determine any abnormality:

‘A good thing for that one might be something along the lines of what you have on CT where you actually have density measurements.’ (PTCP 6M)

Lastly, within this theme, the participants indicated that the CAD tool should have the ability to capture findings, annotate observed patterns and, where applicable, have programmed annotations (a database) that allow comparisons:

‘I think the other thing since it’s used for teaching purposes, it needs to be something that also allows us to capture the findings or the patterns recognised in the image, there should be that portion where you can record it so that even next time when you had a certain image from me you find the same patterns, you can compare to see, are they of the same appearance ‘cause you *are* relying on appearance.’ (PTCP 2M)

### Theme 3: A tool that encourages self-directed learning using a wide variety of pathological conditions would be ideal

The participants highlighted the importance of a CAD tool that encourages self-directed learning for students. For example, they deemed that having a database of various common chest pathologies would be ideal for self-directed learning. Such a database could be used for reference purposes when teaching students so that they could access previous images with relevant information:

‘The other thing that I also wanted to mention is the … I don’t know how possible this could be but obviously it’s also user-dependent. The calibration of the various images attached to various pathologies or various patterns whereby you could have an example of a pneumothorax so that if you have a very new person coming in that you also teaching, they can easily go under the profile of pneumothorax and see pneumothorax looks like this, therefore whatever they see, it’s suggestive of a pneumo or …’ (PTCP 2M)

‘Like a reference type of thing.’ (PTCP 4F)

‘A reference, yes, a reference type of …’ (PTCP 2M)

Furthermore, the participants viewed accession to previous records an important component of a CAD tool:

‘The previous records should be easily … easy accessible.’ (PTCP 4F)

The participants also highlighted the importance of having voice prompts and visual stimulations as part of enhancing independent learning for students:

‘Built-in audio facility. How we gonna do, I don’t know.’ (PTCP 3M)

‘It will communicate with you, telling you start there or start there or have you done this or …’ (PTCP 4F)

‘So, ja, a guiding voice?’ (PTCP 2M)

‘… [*P*]ictures and audio and colours, they really help whatever that you get like if I were to say let’s say maybe if I can maybe say … what can I say? Let’s say metal. I can look at the metal at this point and say I know this is a metal but for a first year … if you can look and listen to the metal somewhere it’s got to capture accurately. Other one you can say metal and put a colour on it, so you grab three things so if you lose one, you *are* left with two. So it’s …’ (PTCP 3M)

‘Visual stimulation.’ (PTCP 4F)

‘Yes.’ (PTCP 3M)

‘Audio stimulation.’ (PTCP 4F)

Chen et al. (2017) assert that implicit knowledge, such as pattern recognition, is gained when learning takes place through the development of skills acquired through the process of repetitive stimulus-response binding. Shanahan (2016) adds that when appropriate approaches are used, students can repeat activities until satisfied with the results. Thus, the developed CAD tool should be able to enhance self-directed learning as part of the ‘repetitive stimulus-binding’ approach to learning, that is incorporate database of various common chest pathologies, voice prompts and visual stimulations which are suitable for technology-based teaching and learning approaches.

## Limitations of the study

This study comprised a small group of participants from one training university; however, their expertise and demographics were appropriate for this study. Consensus was obtained at each point, indicating appropriate relevance to radiography training in general. However, the results could not be generalised because the study was limited to one institution only.

## Conclusion

This study identified the key needs for and capabilities of CAD tools essential to be considered by MI educators for designing tools aimed at enhancing the development of implicit skills. It is desired that the design of CAD tools using identified keys would assist in enhancing the teaching of chest pattern recognition skills at the undergraduate level.
